# Association between traffic-related air pollution exposure and fertility-assisted births

**DOI:** 10.1088/2752-5309/accd10

**Published:** 2023-04-26

**Authors:** Daphne Thampy, Verónica M Vieira

**Affiliations:** 1 Department of Environmental and Occupational Health, Program in Public Health, University of California, Irvine, CA, United States of America; 2 Lewis Katz School of Medicine, Temple University, Philadelphia, PA, United States of America

**Keywords:** air pollution, infertility, fecundity, assisted reproductive technology

## Abstract

Previous studies have suggested that traffic-related air pollution is associated with adverse fertility outcomes, such as reduced fecundability and subfertility. The purpose of this research is to investigate if PM_2.5_ exposure prior to conception or traffic-related exposures (traffic density and distance to nearest major roadway) at birth address is associated with fertility-assisted births. We obtained all live and still births from the Massachusetts state birth registry with an estimated conception date between January 2002 through December 2008. All births requiring fertility drugs or assisted reproductive technology were identified as cases. We randomly selected 2000 infants conceived each year to serve as a common control group. PM_2.5_ exposure was assessed using 4 km spatial satellite remote sensing, meteorological and land use spatiotemporal models at geocoded birth addresses for the year prior to conception. The mean PM_2.5_ level was 9.81 *µ*g m^−3^ (standard deviation = 1.70 *µ*g m^−3^), with a maximum of 14.27 *µ*g m^−3^. We calculated crude and adjusted fertility treatment odds ratios (ORs) and 95% confidence intervals (CI) per interquartile range of 1.72 *µ*g m^−3^ increase in PM_2.5_ exposure. Our final analyses included 10 748 fertility-assisted births and 12 225 controls. After adjusting for parental age, marital status, race, maternal education, insurance status, parity, and year of birth, average PM_2.5_ exposure during the year prior to conception was weakly associated with fertility treatment (OR: 1.01; 95% CI: 0.97, 1.05). Fertility-assisted births were inversely associated with traffic density (highest quartile compared to lowest quartile, OR: 0.92; 95% CI: 0.83, 1.02) and positively associated with distance from major roadway (OR per 100 m: 1.01; 95% CI: 1.00, 1.02) in adjusted analyses. We did not find strong evidence to support an adverse relationship between traffic-related air pollution exposure and fertility-assisted births.

## 1. Introduction

Previous studies have observed associations between air pollution and various fertility related outcomes, including subfertility, a condition that affected 11% of women in the United States between 2006 and 2010 [1]. Subfertility is defined as the reduced ability to conceive after 1 year of unprotected intercourse during ovulation [2]. As more couples have faced issues with fertility, many have opted for fertility enhancing drugs or assisted reproductive technology (ART), such as *in vitro* fertilization (IVF) or gamete intrafallopian transfer. In 2018, the most recent year of available data, 203 119 ART procedures were reported to the CDC in which 99% were IVF procedures [3]. Major causes of subfertility have been categorized as ovulation disorders, male reproductive disorders, fallopian tube factors, unknown, and other causes [4].

Traffic-related air pollution is one potential risk factor that has been linked to fertility [4, 5]. PM_2.5_, a type of traffic-related particulate matter with a diameter of 2.5 *µ*m or less, is formed as a result of the combustion of fuels and has been specifically found to be associated with various subfertility outcomes. In animal studies, when examining the effects of ambient pollution from automobile traffic, researchers observed decreased number of newborns, increased embryo implantation failure rate, decreased fertility index (number of pregnant females/total number of females), and increased spontaneous abortion rates in mouse models [6, 7]. Exposure to PM_2.5_ has been shown to decrease antral follicle count in females exposed to PM_2.5_ [6]. The lower antral follicle count may be due to disruption of endocrine activity or oxidative stress [8]. A cross-sectional study of male Taiwanese participants investigated the specific health effects of PM_2.5_ on semen quality and observed an association between increased levels of PM_2.5_ exposure and decreased levels of normal sperm morphology with a higher level of sperm concentration [9]. PM_2.5_ exposure may cause DNA damage, mutation, and methylation in males [10].

Previous studies of PM_2.5_ exposure and fertility outcomes have mainly focused on couples undergoing IVF treatments [11–16]. Increased PM_2.5_ exposure has been shown to decrease pregnancy rates during IVF embryo culture [11]. A cohort study of women from Massachusetts General Hospital Fertility Center also found an association between increased PM_2.5_ exposure and lower antral follicle count even after adjusting for age [13]. Other studies have examined how different exposure windows can affect the biological pathways associated with reproductive processes targeted in infertility treatment [14]. Studies of PM_2.5_ exposure and fertility outcomes in the general population include both ecological studies [17, 18] and cohort studies [19–21], which have found inconsistent results. While studies in the U.S. [20, 21] have observed largely null results with PM_2.5_ exposure, studies in Europe and China [17–19] found significant associations between increases in PM_2.5_ exposures and decreases in fecundability [19]. The relationship between fertility outcomes and proximity to major roadways has been generally consistent, with the likelihood of pregnancy increasing with further distance [12, 22, 23].

The purpose of this research is to investigate if PM_2.5_ exposure in Massachusetts prior to conception or traffic-related exposures (traffic density and distance to nearest major roadway) is associated with fertility-assisted births from 2002 to 2008. This study will contribute to our knowledge of fertility outcomes among the general population using highly resolved spatiotemporal estimates of PM_2.5_ exposure. Massachusetts was among the states with the highest ART treatment rates during this time due to state-mandated insurance coverage, making it an advantageous study area for this analysis.

## 2. Methods

### 2.1. Study population

The study population includes all live and still births from the Massachusetts state birth registry with an estimated conception date from 1 January 2002 through 31 December 2008 [24]. Birth records included information about whether ART or fertility drugs were used to conceive. Available birth record data also included geocoded birth address, demographic information for the mother and father, information on pregnancy history and prenatal care, pregnancy risk factors, and birth/delivery characteristics. Among the 533 649 births conceived between 2002 and 2008, 12 328 (2.3%) were conceived with ART or fertility drugs. We randomly selected 2000 infants conceived each year without fertility assistance to serve as the control group of 14 000 among all births. The Institutional Review Boards of the University of California at Irvine and the Massachusetts Department of Public Health approved this research.

### 2.2. Exposure assessment

Satellite remote sensing data was used to assess PM_2.5_, which allowed for spatial coverage throughout Massachusetts and exposure assignment of 95% of Massachusetts births for this study. PM_2.5_ levels were measured daily and averaged for each exposure assessment time interval. The exposure model is described in detail by Girguis *et al* [24]. To summarize, a linear mixed methods model was created using meteorological and land use data as the independent variables and aerosol optical depth (AOD) and 24 h Environmental Protection Agency PM_2.5_ measurements were used as the dependent variable. We obtained 4 km AOD data from the National Oceanic and Atmospheric Administration National Environmental Satellite, Data, and Information Service. The model accounted for the temporally varying relationship between PM_2.5_ and AOD after adjusting for the following confounders: relative humidity, wind speed, elevation, major roads, forest cover, and point emissions. The spatially varying relationship between PM_2.5_ and AOD across Massachusetts was also accounted for by using continuous source estimates for each parameter at each location. Both of these relationships were then used to find daily PM_2.5_ estimates where AOD estimates are not known. The ten-fold cross validation (CV) mean squared prediction errors ranged from 2.42 to 3.50 *μ*g m^−3^, and the CV R^2^ ranged from 0.78 to 0.88, indicating a good performance of the model [24]. Although traffic is the primary source of PM_2.5_ in the study area, the PM_2.5_ exposure also includes contributions from non-traffic-related sources such as facility emissions. In our primary analysis, average PM_2.5_ exposure was assessed for each birth 1 year prior to conception using the address on the birth record [21]. Additionally, those missing over 70% of air pollution data were excluded, which included 1580 fertility-assisted births. Among the controls, 1745 infants were excluded due to missing or insufficient PM_2.5_ data.

Additionally, to further understand the spatial relationship between traffic-related pollution and ART, distance to the nearest major roadway and traffic density were calculated based on birth location. Road segments and traffic density were obtained from the MA Department of Transportation. For the distance to nearest major roadways, the shortest distance between each Class I (limited access highways) or Class II (multilane highways without limited access) road type and each geocoded birth address were calculated in meters using geographic information system software (ArcGIS). The traffic density was determined by summing available annual average daily traffic (AADT) for both road types using a 200 m radius of each road segments within each class. Nearest available AADT levels were used for segments with missing AADT data.

We also conducted an additional sensitivity analysis. As couples with difficulty conceiving usually have tried a year or more before opting for fertility treatment [2], we examined the association between fertility-assisted births and average exposure 2 years prior to conception12 as this may reflect a relevant exposure window as well [12].

### 2.3. Statistical analyses

After exposure was assigned, logistic regression models were used to calculate crude odds ratios (ORs), adjusted odds ratios, and 95% confidence intervals (CIs) for the association between fertility-assisted births and a corresponding interquartile range (IQR) increase of PM_2.5_ exposure. We fit separate models for traffic density modeled categorically by quartiles and continuous distance to roadways. Variables from the birth record data that were significantly associated with the outcome in univariate models were retained for inclusion in the final multivariable model. Analyses of complete data were adjusted for maternal and infant characteristics available in the birth record: mother and father’s age, marital status, parity, maternal race/ethnicity and education level, insurance type, birth year of infant, and adequacy of care. All analyses were conducted using R (R Software Version 4.0.5).

## 3. Results

### 3.1. Summary of birth characteristics

Massachusetts birth records were obtained for 12 255 controls and 10 748 cases who needed assisted reproductive fertility treatment or drugs conceived from 1 January 2002 to 31 December 2008. During this time, the mean PM_2.5_ level 1 year prior to conception was 9.81 *µ*g m^−3^ (standard deviation = 1.70 *µ*g m^−3^), with IQR of 1.72 *µ*g m^−3^ and a maximum of 14.27 *µ*g m^−3^. The mean PM_2.5_ level 2 years prior to conception was 10.43 *µ*g m^−3^ (standard deviation = 1.85 *µ*g m^−3^), with IQR of 1.87 *µ*g m^−3^ and a maximum of 20.49 *µ*g m^−3^. Maps of annual average PM_2.5_ exposure (*µ*g m^−3^) for calendar years 2002–2008 show that exposure declined over time and was highest in the eastern part of the state (supplemental figure 1).

As expected, cases were more likely to be older mothers; over half (52.7%) were 35 years or older compared to only 22.1% of control mothers (table 1). About 87% of case mothers were of non-Hispanic White race/ethnicity, compared to 68% of control mothers. Case mothers were also more likely to be nulliparous, have private insurance, and be more educated. They were also generally healthier, as only 1.2% of case mothers reported smoking during pregnancy compared to 7.3% of control mothers. Case mothers were also more likely to receive adequate prenatal care (87%) compared to control mothers (77%).

**Table 1.** Characteristics of fertility-assisted birth cases and randomly selected controls in Massachusetts, conceived 2002–2008.

*n* (%) ^a^	Controls (*n* = 12 255)	Cases (*n* = 10 748)
**Infant sex**		
Male	6253 (51.0)	5575 (51.9)
Female	6002 (49.0)	5173 (48.1)
**Maternal age**		
<20 years	768 (6.3)	10 (0.1)
21–24 years	1994 (16.3)	107 (1.0)
25–29 years	2925 (23.9)	1182 (11.0)
30–34 years	3860 (31.5)	3781 (35.2)
35 + years	2708 (22.1)	5668 (52.7)
**Paternal age**		
<20 years	254 (2.1)	2 (0.01)
21–24 years	1128 (9.2)	44 (0.4)
25–29 years	2079 (17.0)	678 (6.3)
30–34 years	3461 (28.2)	2914 (27.1)
35 + years	4129 (33.7)	6555 (61.0)
Unknown	1204 (9.8)	555 (5.2)
**Marital status**		
Married	8276 (67.5)	10 176 (94.7)
Not married	3979 (32.5)	572 (5.3)
**Parity**		
0	5451 (44.5)	5427 (50.5)
1	4273 (34.9)	3740 (34.8)
2–10	2508 (20.5)	1573 (14.6)
Unknown	23 (0.2)	8 (0.1)
**Adequacy of prenatal care**		
Adequate	9464 (77.2)	9352 (87.0)
Intermediate	2240 (18.3)	1206 (11.2)
Inadequate	406 (3.3)	121 (1.1)
Unknown	125 (1.0)	66 (.6)
None	20 (0.2)	3 (0.0)
**Smoking during pregnancy**		
Yes	892 (7.3)	130 (1.2)
No	11 363 (92.7)	10 618 (98.8)
**Drinking during pregnancy**		
Yes	227 (1.9)	142 (1.3)
No	12 013 (98.0)	10 592 (98.5)
Unknown	15 (0.1)	14 (0.1)
**Gestational age**		
Less than 37 weeks	957 (7.8)	3509 (32.6)
More than or equal to 37 weeks	11 287 (92.1)	7239 (67.4)
Unknown	11 (0.1)	—
**Small for gestational age**		
Yes	1289 (10.5)	1977 (18.4)
No	10 944 (89.3)	8721 (81.1)
Unknown	22 (0.2)	50 (0.5)
**Maternal race/Ethnicity**		
Non-Hispanic White	8324 (67.9)	9318 (86.7)
Non-Hispanic Black	1022 (8.3)	294 (2.7)
Hispanic	1739 (14.2)	371 (3.5)
Asian/Pacific Islander	901 (7.4)	651 (6.1)
Other	261 (2.1)	105 (1.0)
Unknown	8 (0.1)	9 (0.1)
**Maternal education**		
<12th grade	1317 (10.7)	75 (0.7)
High school graduation	3211 (26.2)	1080 (10.0)
Some college	7709 (62.9)	9570 (89.0)
Unknown	18 (0.1)	23 (0.2)
**Maternal language preference**		
English	10 762 (87.8)	10 372 (96.5)
Spanish	57 (0.5)	16 (0.1)
Portuguese	338 (2.8)	35 (0.3)
Other	1059 (8.6)	298 (2.8)
Unknown	39 (0.3)	27 (0.3)
**Delivery source of payment**		
Blue Cross	499 (4.1)	638 (5.9)
Commercial	637 (5.2)	616 (5.7)
HMO	6847 (55.9)	9129 (84.9)
Medicaid/Commonwealth	3215 (26.2)	257 (2.4)
Other Government	897 (7.3)	72 (0.7)
Other	153 (1.2)	34 (0.3)
Unknown	7 (0.1)	2 (0.0)
**Year**		
2002	2000 (16.3)	1775 (16.5)
2003	569 (4.6)	565 (5.3)
2004	1837 (15.0)	1906 (17.7)
2005	1966 (16.0)	1828 (17.0)
2006	1943 (15.9)	1727 (16.1)
2007	1966 (16.0)	1391 (12.9)
2008	1974 (16.1)	1556 (14.5)
**Traffic density**		
0–12.57 AADT	1971 (16.1)	2521 (23.5)
12.58–44.01 AADT	3574 (29.2)	3348 (31.1)
44.02–144.63 AADT	3783 (30.9)	2627 (24.4)
144.64–1603 AADT	2927 (23.9)	2252 (21.0)
**Distance to major roadways;** (m) mean (standard deviation)	263.7 (389.7)	335.0 (445.4)
**PM_2.5_ 1 year prior to conception;** *µ*g m^−3^ mean (standard deviation)	9.9 (1.8)	9.8 (1.6)
**PM_2.5_ 2 years prior to conception;** *µ*g m^−3^ mean (standard deviation)	10.5 (1.9)	10.4 (1.8)

Abbreviations: AADT, annual average daily traffic; CI, confidence interval; OR, odds ratio; PM_2.5_, particulate matter with a diameter of 2.5 *µ*m or less.

^a^ Percentages may not sum to 100% due to rounding.

### 3.2. Air pollution analyses

Our primary analysis examined associations with PM_2.5_ exposure during the year prior to conception. An increase of IQR = 1.72 *µ*g m^−3^ PM_2.5_ was inversely associated with fertility-assisted births in unadjusted analyses (OR: 0.95; 95% CI: 0.93, 0.98; supplemental table S1). After adjusting for parental ages, marital status, maternal race/ethnicity, maternal education, insurance status, parity, year of birth, and adequate prenatal care, PM_2.5_ exposure was weakly associated with fertility treatment (OR: 1.01; 95% CI: 0.97, 1.05; table 2). We observed similar results for average PM_2.5_ exposure 2 years prior to conception (OR: 1.01; 95% CI: 0.96, 1.07 per IQR = 1.87 *µ*g m^−3^; supplemental table S2).

**Table 2.** Adjusted odds ratios^a^ and 95% confidence interval for exposure to PM_2.5_ in Massachusetts, and fertility-assisted births conceived between 2001–2008.

Predictor variables	Adjusted ORs (95% CI)
**PM_2.5_ (per IQR = 1.72 *µ*g m^−3^) 1 year prior to conception**	1.01 (0.97, 1.05)
**Maternal age**	
<20 years	Referent
21–24 years	0.86 (0.42, 1.77)
25–29 years	2.04 (0.99, 4.21)
30–34 years	3.18 (1.54, 6.56)^b^
35 + years	6.11 (2.96, 12.64)^b^
**Paternal age**	
<20 years	Referent
21–24 years	2.43 (0.54, 10.95)
25–29 years	4.67 (1.04, 21.02)^b^
30–34 years	5.75 (1.27, 25.91)^b^
35 + years	7.88 (1.75, 35.55)^b^
**Marital status**	
Married	Referent
Not married	0.33 (0.29, 0.39)^b^
**Parity**	
0	Referent
1	0.64 (0.60, 0.69)^b^
2–10	0.46 (0.42, 0.51)^b^
**Adequacy of prenatal care**	
Adequate	Referent
Intermediate	0.91 (0.83, 1.00)
Inadequate	0.61 (0.48, 0.79)^b^
None	1.21 (0.82, 1.80)
Unknown	0.92 (0.17, 4.95)
**Maternal race/Ethnicity**	
Non-Hispanic White	Referent
Non-Hispanic Black	0.65 (0.55, 0.76)^b^
Hispanic	0.88 (0.75, 1.03)
Asian/Pacific Islander	0.65 (0.57, 0.73)^b^
Other	0.75 (0.56, 0.99)^b^
**Maternal education**	
<12th grade	Referent
High school graduation	1.64 (1.23, 2.18)^b^
Some college	1.92 (1.45, 2.54)^b^
**Delivery source of payment**	
Blue Cross	Referent
Commercial	0.89 (0.74, 1.06)
HMO	1.04 (0.91, 1.19)
Medicaid/Commonhealth	0.29 (0.24, 0.35)^b^
Other Government	0.27 (0.20, 0.36)^b^
Other	0.28 (0.17, 0.44)^b^
**Birth year**	
2002	Referent
2003	1.17 (1.00, 1.38)
2004	1.18 (1.06, 1.32)^b^
2005	1.13 (1.01, 1.27)^b^
2006	1.11 (0.99, 1.24)
2007	0.91 (0.80, 1.03)
2008	1.01 (0.90, 1.15)

Abbreviations: CI, confidence interval; IQR, interquartile range; OR, odds ratio; PM_2.5_, particulate matter with a diameter of 2.5 *µ*m or less.

^a^ Adjusted for parental age, marital status, maternal race/ethnicity, maternal education, insurance status, parity, and year of birth.

^b^ Statistically significant association at alpha = 0.05.

We also examined associations with other measures of traffic-related air pollution in secondary analyses and observed similar movement of associations towards the null after adjustment. Increased distance from birth address to the nearest major roadway was associated with higher odds of fertility-assisted births in unadjusted analyses (OR: 1.04; 95% CI: 1.04, 1.05 per 100 m increased distance; supplemental table S1). After adjustment for covariates, the association between distance to nearest major roadway and fertility-assisted births was attenuated (OR: 1.01; 95% CI: 1.00, 1.02 per 100 m increased distance; supplemental table S3). Traffic density was inversely associated with fertility treatment in the unadjusted and adjusted analyses for every quartile increase in AADT (supplemental tables S1 and S3). The adjusted OR for the highest quartile of traffic density associated with fertility-assisted births compared to the lowest quartile was 0.92 (95% CI: 0.83, 1.02).

## 4. Discussion

In adjusted analyses, we observed weak positive associations between the odds of fertility-assisted births and increased PM_2.5_ exposure in Massachusetts from 2001 to 2008. For other traffic related exposure measures, associations were protective. ORs were less than one for higher traffic density and increased with greater distance to major roadways. PM_2.5_ measures represent larger scale pollution whereas traffic density and distance to major roads represent more local measures of pollution [24]. The fact that fertility-assisted births were associated with PM_2.5_, but not local measures of traffic-related air pollution, may be indicative of incomplete adjustment due to individual level SES or access to fertility treatment centers. Although other studies have seen that greater distance from a major roadway was associated with better fertility outcomes [12, 22, 23], the observed discrepancies in associations may be due to differences in the housing, noise levels, and neighborhood environment.

The PM_2.5_ exposure results are in agreement with some studies [12, 14, 20]. For example, Boulet *et al* looked at associations between IVF treatment outcomes and average daily concentrations of PM_2.5_ in the United States and found no association between PM_2.5_ and implantation, pregnancy, and live births [14]. Similarly, in a study of 500 American couples, Nobles *et al* did not find strong evidence of an association between PM_2.5_ and fecundability (OR: 1.02, 95% CI: 0.80, 1.28) [20]. Mahalingaiah *et al* investigated infertility risk by PM exposure from September 1993 to December 2003, among 36 294 women in the Nurses’ Health Study II, and found similar mixed results for primary infertility and PM_2.5_. (Hazard Ratio (HR): 0.94; 95% CI: 0.79, 1.13) and distance to major roadway <200 m (HR: 1.05; 95% CI: 0.94, 1.17) [12].

It is possible that PM_2.5_ levels in Massachusetts are not high enough to observe adverse subfertility associations. The mean PM_2.5_ level 1 year prior to conception was 9.81 *µ*g m^−3^ with an IQR of 1.72 *µ*g m^−3^ and a maximum of 14.27 *µ*g m^−3^. A study in Barcelona, Spain [17], observed an increase in PM_2.5_ (IQR = 2.51 *µ*g m3) resulted in a reduced fertility rate of 0.98 (95% CI: 0.95, 1.02). This study had PM_2.5_ levels that were about twice the levels seen in Massachusetts (mean 17.12 *µ*g m^−3^ vs. and maximum 23.48 *µ*g m^−3^). Barcelona has some of the highest air pollution exposure in Europe partially due to high traffic density, high proportion of diesel-powered cars, high population density, and low precipitation [17]. A similar study [18] with high levels of PM_2.5_ in China (average PM_2.5_ was 48 *µ*g m^−3^ with a max of 82 *µ*g m^−3^) found a 2.0% decrease in county-level fertility rates with every 10 *µ*g m^−3^ increase of PM_2.5_. A study by Li *et al*, also in China (average PM_2.5_ was 50 *µ*g m^−3^ with a max of 94 *µ*g m^−3^), found significantly reduced fecundity ORs of 0.89 (95% CI: 0.86, 0.92) for every 10 *µ*g m^−3^ increase of PM_2.5_ 1 year prior to conception [21]. Both Li *et al* [21] and Nobles *et al* [20] posited that there could possibly be a threshold for the chronic effects of PM_2.5_ exposure with fecundity. It is also possible that the composition of PM_2.5_ in China and Barcelona differs in its toxicity compared to that in the United States.

An additional consideration is the timing of exposure. We assessed exposure as the average for one and 2 years prior to conception as had been done in previous cohort studies [12, 21], but other exposure windows may be more biologically relevant. A retrospective birth cohort study, which measured exposures (SO_2_, NO_2_, PM_2.5_, O_3_, PAH) from a central monitoring station during the 2 month period before the first menstrual cycle with unprotected sex, found a significant association between an increase in exposure of averaged PM_2.5_ levels and a short-term decrease in fecundability by about 22% in Czech Republic [19]. Another study examined how different exposure windows can affect the biological pathways associated with reproductive processes targeted in infertility treatment [15]. After estimating PM_2.5_ exposure in the 1, 2, and 3 d, 2 weeks, and 3 months prior to blood collection during ovarian stimulation, they found several pro-inflammatory, anti-inflammatory, and metabolic pathways associated with long-term PM_2.5_ exposure (2–3 months) which were not as stimulated within acute exposure windows. However, subacute exposure of PM_2.5_ using an exposure window of 3 d before embryo transfer in women undergoing IVF treatment living in Barcelona was associated with increased negative outcomes including achieving no pregnancy through IVF [16].

This study has several strengths, including a large sample size, linkage to ART information, and the use of a validated spatiotemporal satellite PM_2.5_ model for Massachusetts. This finer resolution exposure assessment provided more precise air pollution measures than monitoring stations alone. We also included traffic density and distance to nearest major roadway to further investigate traffic-related air pollution. The large number of births requiring fertility assistance in our analyses allowed us to analyze 7 years of data during a time period where fertility treatments were recorded on Massachusetts birth certificates.

The analysis also has some limitations. Exposure was assessed at the residential address on the birth certificate and pregnant couples are a mobile population. This may have biased results if couples with fertility-assisted births had different mobility than couples without assisted births. Information on indoor PM_2.5_ levels and time spent indoors versus outdoors also was not available. Another limitation is that confounders were restricted to variables available on the birth certificates. Although we controlled for maternal education and insurance status which are measures of individual level socioeconomic status, we were lacking direct measures of household income. Lastly, our outcome of subfertility is defined as births requiring the use of ART or fertility, which may not be comparable to other measures of infertility and fecundity in terms of associations with PM_2.5_. However, the fact that there was state-mandated insurance coverage of fertility treatments in Massachusetts with reporting on birth records makes this a novel cohort to study.

## 5. Conclusions

This study did not find strong evidence to support an adverse relationship between traffic-related air pollution exposure and fertility-assisted births. We examined PM_2.5_ exposure, traffic density, and distance to nearest major roadway for over 10 000 fertility-assisted births in Massachusetts using birth records with mandated reporting of ART and fertility drug utilization. Our findings suggested that increased PM_2.5_ levels may be positively associated with subfertility among couples who were able to have successful births, but results were not statistically significant and distance to nearest roadway and traffic density were both inversely associated. These inconsistent results may be due to the generally lower PM_2.5_ levels observed in Massachusetts. Further studies in more highly exposed study areas are needed to better understand this.

## Data Availability

The exposure data used in this study are available upon reasonable request from the authors. Health data used in this study are only available by contacting Massachusetts Department of Public Health to obtain access to the birth records data. The data cannot be made publicly available upon publication because they are owned by a third party and the terms of use prevent public distribution. The data that support the findings of this study are available upon reasonable request from the authors.
